# Deiodinase 3 Deficiency Impairs Hippocampal and Lateral Ventricle Neurogenesis in Middle-Aged Female Mice

**DOI:** 10.3390/ijms27177607

**Published:** 2026-08-25

**Authors:** Zhaofei Wu, M. Elena Martinez, Arturo Hernandez

**Affiliations:** 1 MaineHealth Institute for Research, MaineHealth, Scarborough, ME 04074, USA; 2 Graduate School of Biomedical Science and Engineering, University of Maine, Orono, ME 04469, USA; 3 Department of Medicine, Tufts University School of Medicine, Boston, MA 02111, USA

**Keywords:** adult neurogenesis, thyroid hormone, deiodinase 3 (DIO3), subgranular zone (SGZ), subventricular zone (SVZ), sexual dimorphism

## Abstract

Thyroid hormone (TH, the active metabolite T_3_)) plays a critical role in neuronal proliferation and differentiation during development. However, its role in adult neurogenesis (ANG) remains incompletely understood. To gain insight into the role of T3 in ANG, we used a mouse model (*Dio3*KO mouse) with a deficiency in type 3 deiodinase, which clears T3 in the brain. We observed that the number of DCX/RBFOX3 double-positive cells is significantly reduced in the subgranular zone (SGZ) and the subventricular zone (SVZ) of 10-month-old female *Dio3*KO mice, suggesting impaired ANG in these neurogenic niches. However, no significant changes were observed in IBA1/GFAP or OLIG2/GFAP double-positive cells, suggesting that the neurogenic deficits are not associated with neuroinflammation, gliosis, or altered oligodendrogenesis. Cortical RNA-seq analysis further identified dysregulated T3-responsive genes implicated in ANG, revealing marked sex-dependent differences in gene expression. Proof-of-concept experiments in type 2 deiodinase 2 (*Dio2*) and *Dio3* double knockout mice revealed a rescue of the neurogenic phenotype, supporting the hypothesis that loss of DIO2-mediated T3 generation counteracts the detrimental effects of DIO3 deficiency on ANG. Collectively, these findings identify DIO3- and DIO2-me fotmdiated T3 homeostasis in neural cells as critical contributors to T3-dependent ANG in females, with implications for neurodegenerative diseases and their sexually dimorphic prevalence.

## 1. Introduction

Adult neurogenesis (ANG) is a biological process responsible for generating new, functional neurons from neural stem/progenitor cells in the adult brain. This process consists of four key stages: cell proliferation (division), differentiation (specialization), migration to final locations, and the establishment of functional neuronal circuits. ANG influences multiple brain functions, including cognition, learning, memory, and social and behavior changes [1]. Critical areas of ANG are the subventricular zone (SVZ) of the lateral ventricles and the subgranular zone (SGZ) in the dentate gyrus [2]. Defects in ANG have been linked to a wide range of psychiatric (autism spectrum disorder, schizophrenia, bipolar disorder, anxiety and major depression etc.) and neurological disorders which include epilepsy, stroke, neurodegenerative diseases (Alzheimer’s, Parkinson’s, Amyotrophic Lateral Sclerosis and Huntington’s disease etc.) [3,4,5,6]. Targeting ANG is considered a promising approach for treating some brain disorders. However, the underlying molecular and cellular mechanisms of ANG are not yet fully understood, and the effectiveness of targeting abnormal ANG for treatment remains very limited [7,8]. Comprehensive elucidation of the mechanisms of ANG may reveal potential therapeutic targets and support the development of new treatment methods for neurological disease.

Thyroid hormones (THs, including the pro-hormone thyroxine or T4, and the active hormone 3,5,3′-triiodothyronine or T3) are key signaling molecules for appropriate brain functions in adult mammals, including humans [9]. One essential role of THs in the mature mammalian brain is to regulate adult neurogliogenic processes. Adequate regulation of T3 action is necessary for the generation of new neuronal and glial progenitors from neural stem cells (NSCs), as well as their final maturation and neuronal fate [10], making this hormone a major contributor to the regulation of these processes [11]. T3 action in the brain is regulated by a dynamic balance between activating type 2 deiodinase (DIO2), which locally converts T4 to the active hormone T3, and the inactivating type 3 deiodinase (DIO3), which clears both T4 and T3, dampening T3 action [12]. Using *Thra*−/− mice (*Thra* encodes TH receptor alpha), *Slc16a2*−/− mice (*Slc16a2* encodes monocarboxylate transporter 8, an integral membrane TH transporter) and T3 treatment on *Pink1*−/− mice (*Pink1* encodes phosphatase and tensin homolog (PTEN)-induced putative kinase 1, and loss-of-function mutations in PINK1 cause autosomal recessive early-onset Parkinson’s disease and contribute to neurodegeneration [13]), previous studies showed that T3 signaling acts as a neurogenic regulator in the SVZ [14] and SGZ [15,16] of adult mice. However, the mechanisms underlying TH-dependent ANG processes have not been comprehensibly addressed [17]. Furthermore, ANG in females remains insufficiently characterized and merits specific investigation considering the sex bias in the prevalence of psychiatric conditions [18,19,20].

Our previous studies have found that regulation of T3 action exhibits sexual differences [21,22,23]. Many neurological and psychiatric disorders occur at higher rates or show different trajectories in females [24]. ANG is also strongly linked to mood regulation, cognition, and stress adaptation [2], yet most historical animal studies used males [9,14,15,25]. Estrogen, progesterone, prolactin, oxytocin, and their fluctuations across the oestrous/menstrual cycle, pregnancy, postpartum, and menopause profoundly influence neural stem cell proliferation, neuronal maturation, and hippocampal plasticity [26,27,28]. Females therefore provide an understudied model for understanding how endogenous hormonal states shape brain regeneration and circuit remodeling across the lifespan. Therefore, female ANG research may reveal mechanisms of resilience and vulnerability unique to reproduction and aging [29]. In addition, investigation of ANG in females may uncover mechanisms underlying maternal cognition, postpartum mood disorders, cognitive aging, and neuroprotection [30,31]. These insights could lead to sex-specific therapies that are currently lacking in neuroscience and regenerative medicine.

Here, we report that ANG is impaired in the hippocampus of female mice with DIO3 deficiency and that this deficit is rescued by concurrent loss of DIO2, highlighting the importance of these determinants of local control of T3 action for ANG in females.

## 2. Results

### 2.1. Decreased ANG in Female Dio3KO Mice

**Figure 1:**
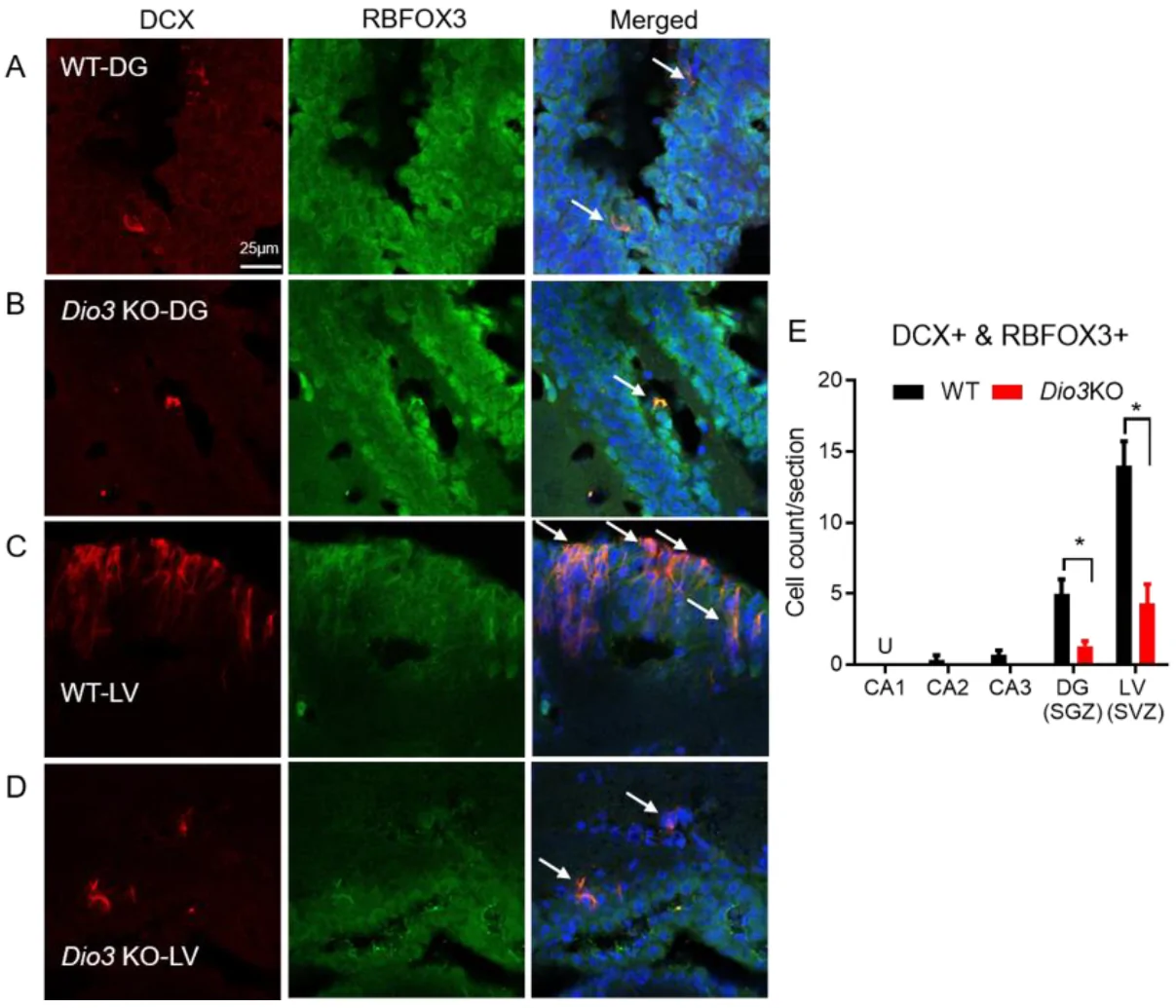
DCX/RBFOX3 double staining in the brain of 10-month-old WT and *Dio3*KO female mice. (**A**,**B**) Representative images of DCX/RBFOX3 double immunofluorescence staining in the subgranular zone (SGZ) of the dentate gyrus. (**C**,**D**) Representative images of DCX/RBFOX3 double immunofluorescence staining in the subventricular zone (SVZ) of the lateral ventricle. DCX staining is shown in red, RBFOX3 (NeuN) staining in green, and DAPI nuclear staining in blue. (**E**) Quantification of DCX/RBFOX3 double-positive cells. U, Undetectable. * *p* < 0.05 by Student’s *t*-test (*n* = 3 WT, 4 KO). CA1-3: three subfields of the Cornu Ammonis (the hippocampus proper); DG, dentate gyrus; LV, lateral ventricle. The arrow indicates a positive cell.

To examine ANG in the female mouse brain, we performed double immunofluorescence staining for the neurogenic marker doublecortin (DCX) [32,33] and the neuronal marker RBFOX3 (NeuN) [34] on brain sections from 10-month-old female Dio3KO mice. We focused our analyses on the SVZ and SGZ, two major neurogenic niches in the adult brain, and observed that the number of DCX/RBFOX3 double-positive cells, representing newly generated neurons, was reduced in both the SGZ (Figure 1A,B) and the SVZ (Figure 1C,D) of female Dio3KO mice compared with wild-type control mice (Figure 1E). We also observed quantification of DCX-positive cells (Figure S1A) and CALB1-positive cells (Figure S1B) in the regions of interest. ANG is largely restricted to the SGZ of the dentate gyrus under physiological conditions in the hippocampus; therefore, CA1–CA3 regions, which are not established neurogenic niches [35], were used as internal negative-control regions.

### 2.2. No Increase in Neuroinflammatory Markers Observed in the Brain of Dio3KO Female Mice

**Figure 2:**
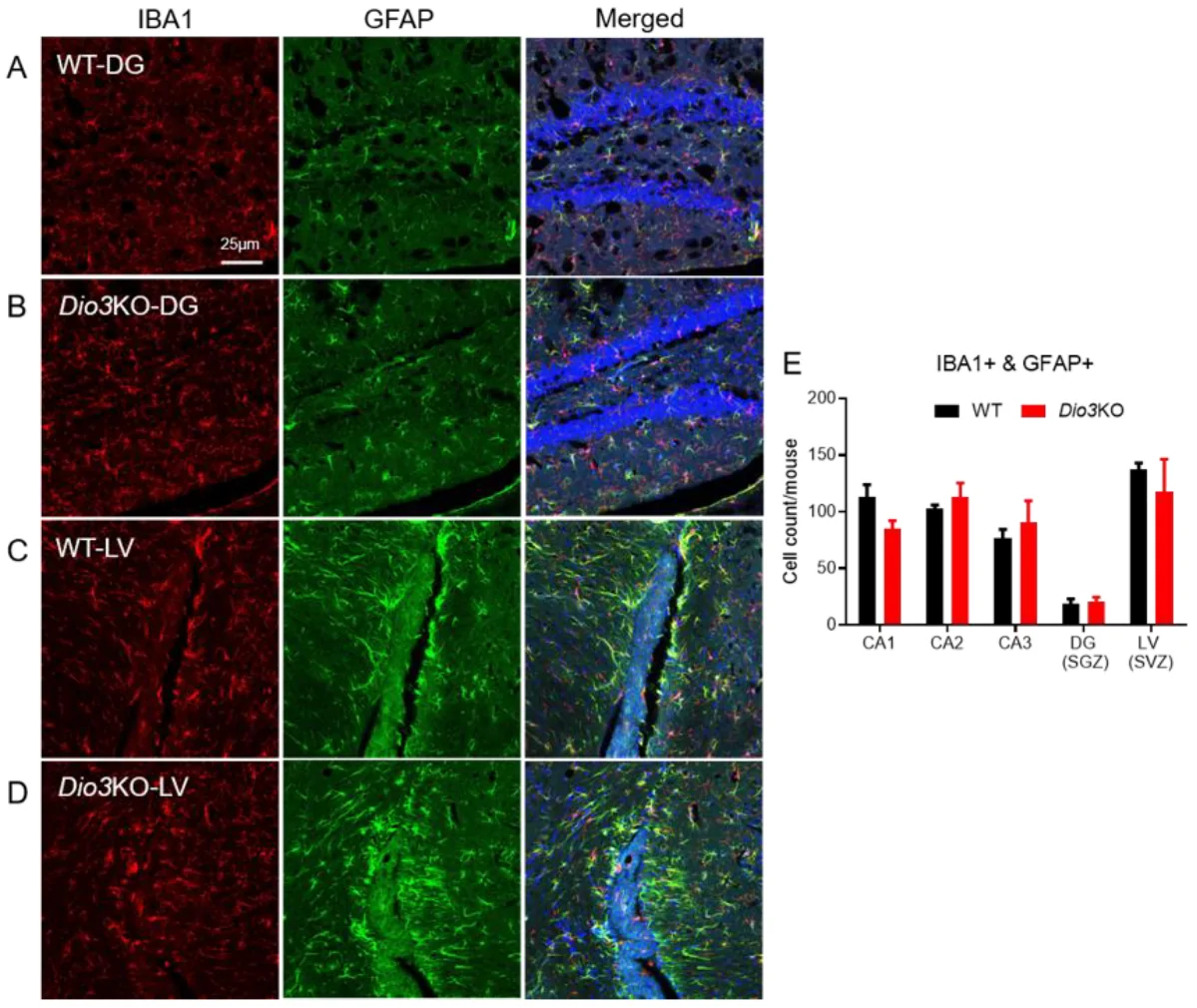
IBA1/GFAP double staining of 10-month-old female brains. (**A**,**B**) Representative images of IBA1/GFAP double immunofluorescence staining in the SGZ of the dentate gyrus. (**C**,**D**) Representative images of IBA1/GFAP double immunofluorescence staining in the SVZ of the lateral ventricle. (**E**) Quantification of IBA1/GFAP double-positive cells. IBA1 staining is shown in red, GFAP staining in green, and DAPI nuclear staining in blue. *n* = 3 WT, 4 KO. CA1-3: three subfields of the Cornu Ammonis (the hippocampus proper); DG, dentate gyrus; LV, lateral ventricle.

To examine potential effect on gliogenesis or neuroinflammation, we performed double immunofluorescence staining for the microglial marker IBA1 [36] and the astrocyte/neural progenitor marker GFAP [37,38] on brain sections from 10-month-old female mice. These results showed that the number of IBA1/GFAP double-positive cells was not altered in either the SGZ of the dentate gyrus (Figure 2A,B) or SVZ of the lateral ventricle (Figure 2C,D) in female *Dio3*KO mice. The absence of an increase in IBA1/GFAP double-positive cells suggests that DIO3 deficiency does not promote substantial microglia–astrocyte reactive interactions or significant neuroinflammatory responses in the brain regions examined (Figure 2E). Quantitative analysis of IBA1 (Figure S2A) and GFAP (Figure S2B) single immunostaining showed no statistically significant differences in the neurogenic regions of interest between female *Dio3*KO mice and controls.

### 2.3. Glial Cell Proliferation Is Not Altered in the Brain of Dio3KO Female Mice

**Figure 3:**
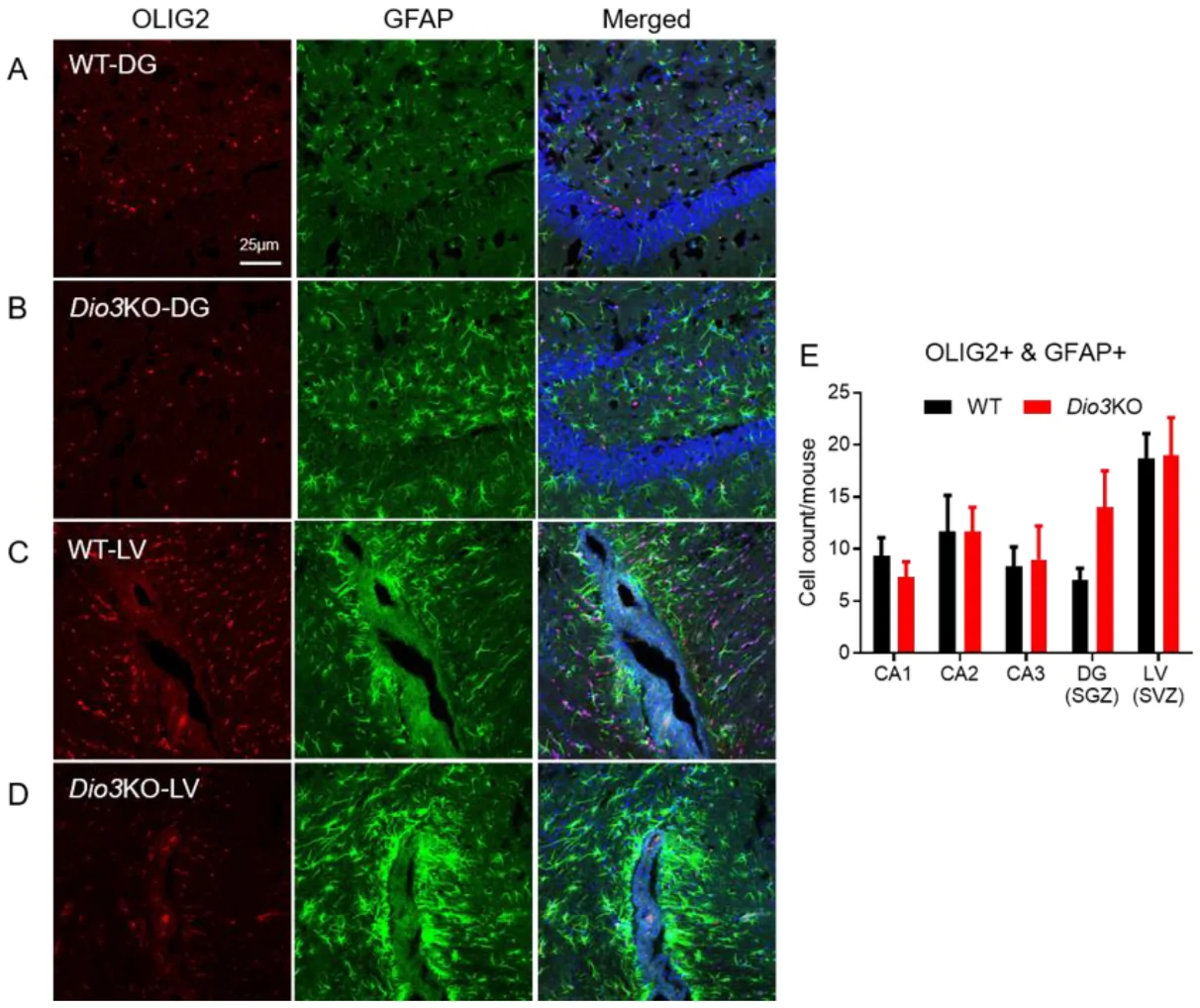
OLIG2/GFAP double staining in the brain of female Dio3KO mice. (**A**,**B**) Representative images of OLIG2/GFAP double immunofluorescence staining in the SGZ of the dentate gyrus. (**C**,**D**) Representative images of OLIG2/GFAP double immunofluorescence staining in the SVZ of the lateral ventricle. (**E**) Quantification of OLIG2/GFAP double-positive cells. OLIG2 staining is shown in red, GFAP staining in green, and DAPI nuclear staining in blue. *n* = 3 WT, 4 KO. CA1-3: three subfields of the Cornu Ammonis (the hippocampus proper); DG, dentate gyrus; LV, lateral ventricle.

To examine any potential effect on oligodendrogenesis, glial progenitor cells or neural stem cells in the female mouse brain, we performed double immunofluorescence staining for the oligodendrocyte marker OLIG2 [39,40] and the astrocyte/neural progenitor marker GFAP on brain sections from 10-month-old female *Dio3*KO mice. These results showed that the number of OLIG2 and GFAP double-positive cells was not altered in either the SGZ of the dentate gyrus (Figure 3A,B), or the SVZ of the lateral ventricle (Figure 3C,D) of female *Dio3*KO mice. The absence of a significant change in OLIG2/GFAP double-positive cells suggests that DIO3 deficiency does not significantly affect the population of putative neural stem/progenitor cells or gliogenic progenitors in the brain regions examined, although additional lineage-specific markers would be required to definitively determine the cellular identity of glial progenitor cells or neural stem cells (Figure 3E).

Quantitative analysis of OLIG2 (Figure S3-1) single immunostaining showed no statistically significant differences in the regions of interest between female *Dio3*KO mice and controls.

BrdU staining in the corpus callosum (CC) of male *Dio3*KO mice did not reveal any significant change in the number of BrdU-positive cells (Figure S3-2). Given that BrdU-labeled cells in the CC have been reported to be predominantly oligodendrocytes derived from progenitor cells originating in the adjacent SVZ [41,42,43], these results suggest that oligodendrocyte production in males is not significantly affected by DIO3 deficiency.

### 2.4. Proof-of-Concept Evidence on ANG in Dio2/Dio3 Double KO Mice

**Figure 4:**
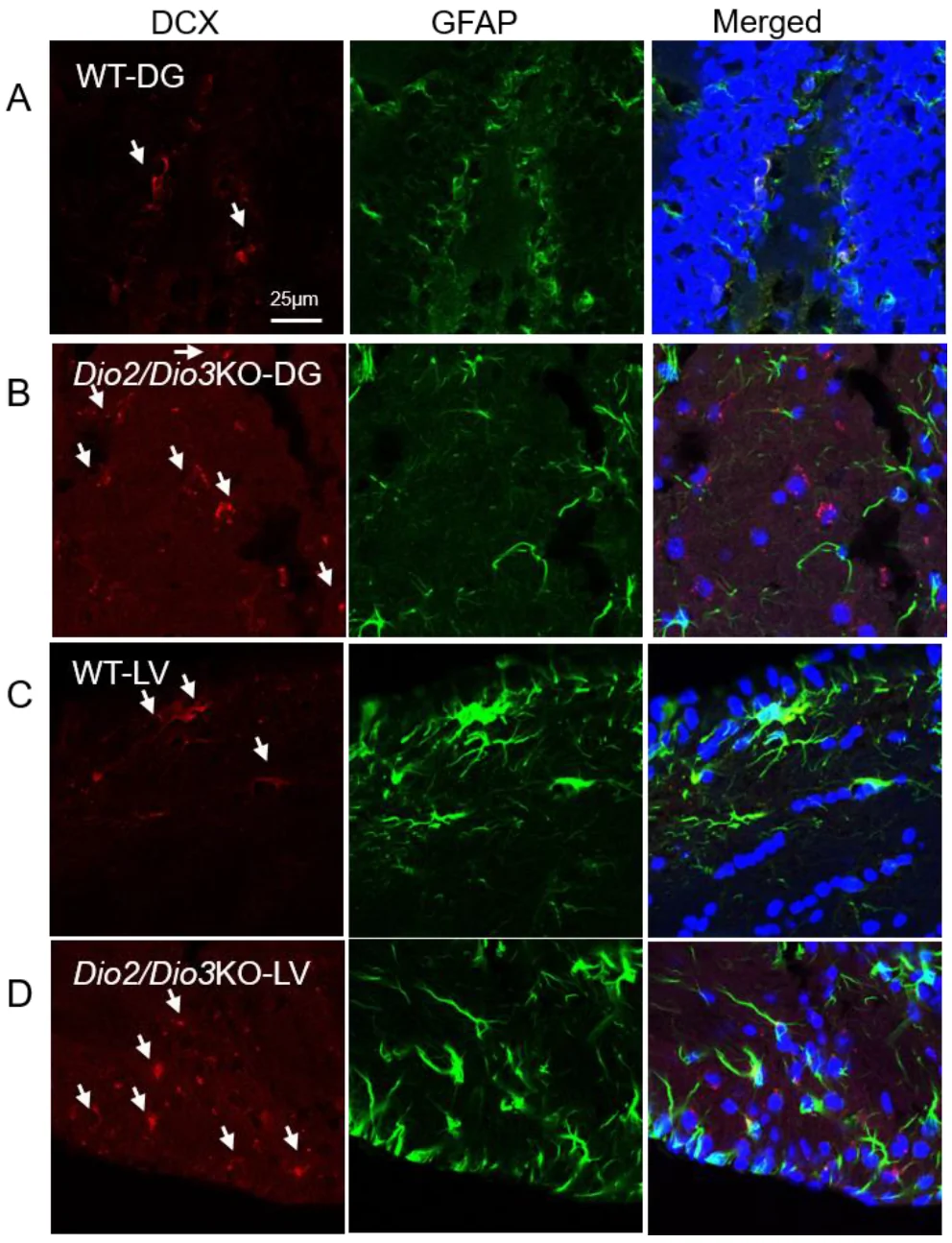
Representative DCX and GFAP double staining in female *Dio2/Dio3* double KO mouse brains. (**A**,**B**) Representative images of DCX/GFAP double immunofluorescence staining in the SGZ of the dentate gyrus. (**C**,**D**) Representative images of DCX/GFAP double immunofluorescence staining in the SVZ of the lateral ventricle. DCX staining is shown in red, GFAP staining in green, and DAPI nuclear staining in blue. DG, dentate gyrus; LV, lateral ventricle. The arrow indicates a positive cell.

DIO2 is a deiodinase with primary physiological function to activate the pro-hormone T4 into the active hormone T3, playing a crucial role in TH homeostatic regulation [44]. To examine the effect of lack of DIO2 function in ANG of Dio3KO females, we performed a proof-of-concept double immunofluorescence analysis using the neurogenic marker DCX and the astrocyte/neural stem cell marker GFAP on brain sections from 10-month-old female Dio2/Dio3 double KO mice. We focused our analyses on SVZ and SGZ in the adult brain, as we had observed that the number of DCX positive cells, representing newly generated neurons, was increased in both the SGZ (Figure 4A,B) and the SVZ (Figure 4C,D) of female Dio2/Dio3 double KO mice compared with control mice. Furthermore, the number of DCX-positive cells was not increased in the SGZ (Figure S4A,B) and increased in the SVZ (Figure S4C,D) of male Dio2/Dio3 double KO mice relative to WT mice. However, GFAP staining did not show an increase in either the SGZ or the SVZ of female (Figure 4) or male (Figure S4) Dio2/Dio3 double KO mice relative to WT mice. These results provide proof of concept that reducing T3 availability through DIO2 deficiency can counteract the T3 overexposure caused by DIO3 deficiency, thereby rescuing the defects in ANG observed in both the SGZ and SVZ.

## 3. Discussion

In the present study, we show that DIO3 deficiency is associated with impaired ANG in the brains of middle-aged female mice. Using DCX/RBFOX3 double immunofluorescence staining, we observed a reduction in newly generated neurons in both the SGZ of the dentate gyrus and the SVZ of the lateral ventricle. These findings identify DIO3 as an important regulator of ANG and suggest that excessive T3 signaling negatively affects neuronal production and maturation within the major neurogenic niches of the adult female mouse brain.

Thyroid hormone is well established as a critical regulator of neural development, influencing neural stem cell proliferation, neuronal differentiation, migration, and survival [12,45,46]. Excessive T3 signaling within brain neurogenic niches can arise independent of circulating thyroid-hormone concentrations. Major molecular drivers include loss of DIO3-mediated T3 inactivation, stabilized or elevated DIO2-dependent T4-to-T3 conversion, dysregulated cellular thyroid-hormone transport, and receptor-level hypersensitivity. Sex-specific hormonal milieus further shape local T3 homeostasis and phenotypic outcomes. Genomic T3 effects in ANG are mediated by thyroid hormone receptor isoforms THRA and THRB. DIO3 is the primary thyroid hormone-inactivating enzyme in the brain, functioning to protect tissues from excessive exposure to T3 [47]. Therefore, DIO3 deficiency in the brain abolishes T3-inactivating function, resulting in elevated T3 exposure within brain tissue. Previous studies have shown that *Dio3*KO mice exhibit increased T3 action in the central nervous system and display behavioral abnormalities including hyperactivity, anxiety-like behaviors, and deficits in social interaction [48,49,50]. The present findings extend these observations by demonstrating that chronic local T3 overexposure is associated with reduced neurogenesis in both the hippocampal and SVZ neurogenic niches of adult female mice.

The reduction in DCX/RBFOX3 double-positive cells suggests impairment at the stage of neuronal differentiation and/or maturation rather than a complete depletion of precursor populations. Consistent with this interpretation, we did not observe significant alterations in GFAP-positive cells or OLIG2/GFAP double-positive cells in either the SGZ or SVZ. In these neurogenic regions, GFAP labels astrocytes as well as radial glia-like neural stem cells, whereas OLIG2/GFAP double-positive cells may represent neural stem/progenitor cells or gliogenic progenitors. The absence of significant changes in these populations suggests that DIO3 deficiency does not substantially affect the abundance of putative neural stem cells or glial progenitors. Instead, excessive thyroid hormone signaling may preferentially disrupt the progression of neural progenitors toward a neuronal fate or impair the survival and maturation of newly generated neurons.

Neuroinflammation is a well-recognized mechanism contributing to impaired ANG in aging and neurological disorders [51,52]. Activated microglia and reactive astrocytes can release pro-inflammatory mediators that suppress neural progenitor proliferation and neuronal differentiation [25,53]. However, we found no significant changes in IBA1/GFAP double-positive cells, nor were there detectable differences in overall IBA1 or GFAP immunoreactivity. These findings suggest that neuroinflammatory responses are unlikely to account for the neurogenic deficits observed in female *Dio3*KO mice. Although subtle alterations in microglial activation states cannot be excluded, our data indicate that impaired ANG occurs independently of major glial inflammatory changes in the regions examined.

Similarly, our results do not support a substantial effect of DIO3 deficiency on oligodendrogenesis. Neither OLIG2-positive cells nor OLIG2/GFAP double-positive cells were significantly altered in the SGZ or SVZ. Furthermore, BrdU labeling in the corpus callosum, a region that receives oligodendrocyte progenitors originating from the adjacent SVZ, did not reveal obvious differences between genotypes. Although these findings need to be comprehensively confirmed, particularly given the limited sample size of the BrdU experiment, they suggest that DIO3 deficiency preferentially affects neuronal rather than oligodendroglial lineage development in the adult brain.

A major finding of this study is the apparent rescue of ANG in *Dio2/Dio3* double knockout mice. As DIO2 catalyzes the conversion of T4 to biologically active T3, the concurrent deficiency in DIO2 is expected to reduce local T3 production and partially counterbalance the excessive thyroid hormone signaling caused by DIO3 deficiency. Consistent with this hypothesis, increased numbers of DCX-positive cells were observed in both the SGZ and SVZ of *Dio2/Dio3* double knockout mice. These findings provide genetic evidence that altered T3 availability is a primary driver of the neurogenic defects observed in DIO3-deficient animals. The rescue experiment therefore supports a causal relationship between excessive T3 signaling and impaired ANG.

The molecular mechanisms underlying this effect remain to be determined. T3 regulates numerous genes involved in neural stem cell maintenance [45], neuronal differentiation [45], synaptic plasticity, and cell-cycle control [54]. Excessive T3 exposure may alter the expression of transcription factors and signaling pathways required for the proper progression of ANG. Notably, cortical RNA-seq analysis preliminarily identified marked sex-dependent alterations in the expression of neurogenesis-related genes, several of which are established thyroid hormone-responsive targets (Table 1), suggesting a potential molecular basis for the sexually dimorphic effects of DIO3 deficiency on adult neurogenesis.

**Figure 5:**
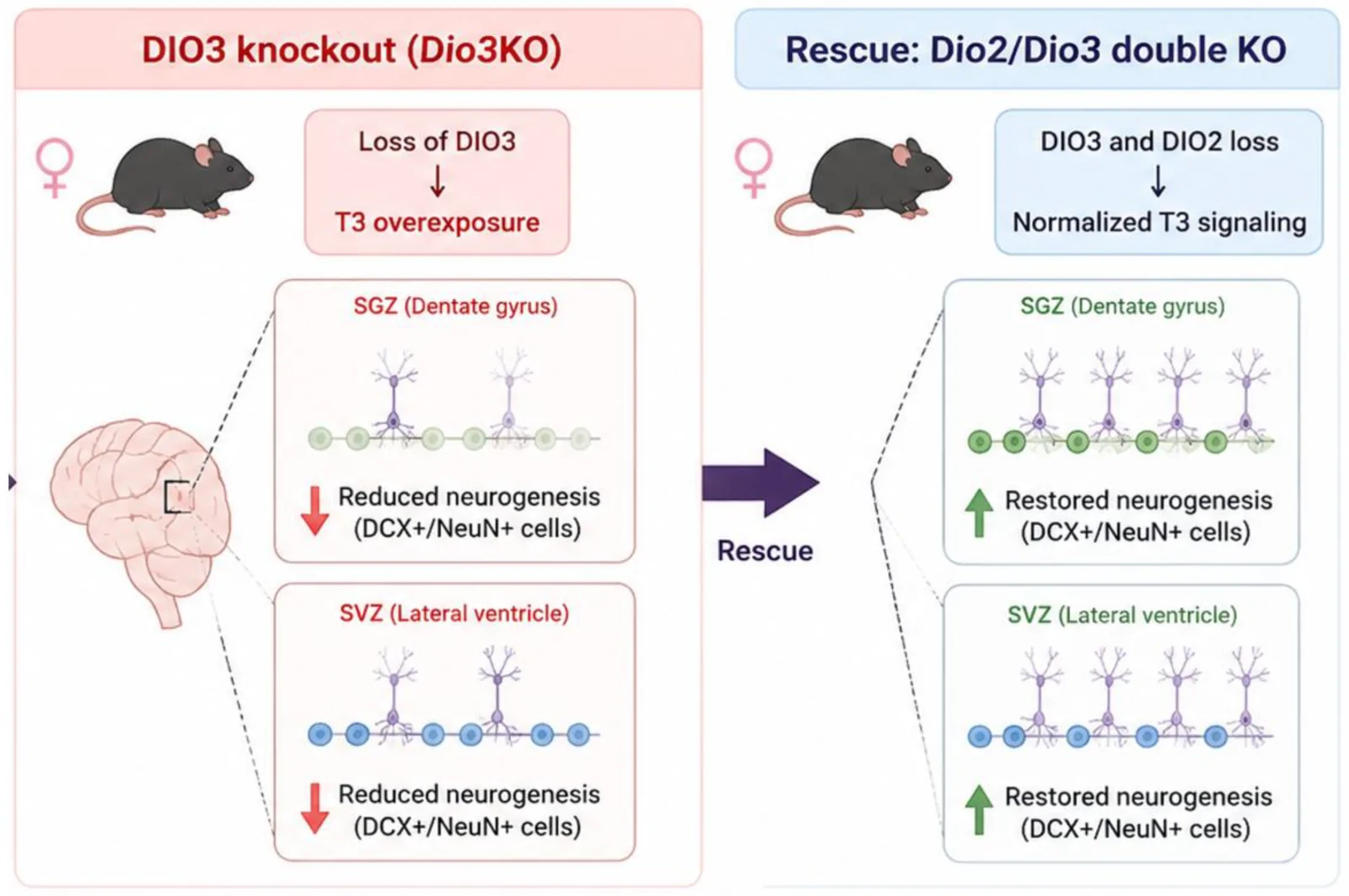
Proposed molecular mechanisms regulating adult neurogenesis in the *Dio3*KO mouse brain. SVZ, subventricular zone of the lateral ventricles; SGZ, subgranular zone of the hippocampal dentate gyrus. DIO3 deficiency causes brain T3 overexposure and impairs adult neurogenesis in the subgranular and subventricular zones of female mice. These defects occur without significant neuroinflammation or altered oligodendrogenesis and are rescued by DIO2 deficiency, identifying DIO3-mediated thyroid hormone homeostasis as a critical regulator of adult neurogenesis.

Integrating evidence from previous studies [30,45,69,70] with our current findings, we propose a molecular model in which altered thyroid hormone regulation of ANG-relevant genes (e.g., *Esr1* and *Foxp1*) contributes to impaired ANG in female *Dio3*KO mice (Figure 5). A limitation of this study is the relatively small number of animals (usually *n* = 3–4 per group), limiting the statistical power of our analyses. Yet, our immunohistochemistry studies indicate ANG impairments in 10-month-old females, an age that represents an inflection point between adult and aged animals. The initial deficits observed at this age are consistently confirmed by in older females (15-month-old). Overall, our findings support future studies integrating lineage tracing, transcriptomic analyses, and cell type-specific manipulations of thyroid hormone signaling to identify the downstream molecular targets responsible for the deficits in ANG caused by abnormal T3 signaling levels.

## 4. Materials and Methods

### 4.1. Experimental Animals

Experimental Dio3KO mice on an out-bred CD1 genetic background used in this study were generated and genotyped as previously described [71]. Dio3 deficient mice on a mixed C57Bl/6J/129Sv/J genetic background were crossed with mice with Dio2 deletion to get Dio2/Dio3 double KO mice [72,73]. We used wildtype (WT) CD1 littermates or WT C57Bl/6J/129Sv/J mice from Ai6 line [74] as controls. The main histological studies to assess ANG were performed in adult animals at ∼10 months of age. At this age, mice may start to exhibit moderate, progressive pathological changes without severe age-related physical deterioration or high mortality, allowing reliable experimental results to be collected and analyzed. RNA sequencing was performed in mice of an older age (15-month-old mice) to better capture transcriptomic alterations that accumulate upon prolonged ageing, which may not yet be robustly detectable at 10 months of age using this technical approach that allows for deep sequencing but may not be informative about changes in particular cell subtypes involved in ANG. Animals were kept under a 12 h light cycle, fed ad libitum in the institutional animal facility. All animal procedures were approved by the Maine Medical Center Research Institute Institutional Animal Care and Use Committee.

### 4.2. Brain Sectioning and Immunofluorescence (IF) Staining

At approximately 10 months of age, mice were anesthetized via intraperitoneal injection of ketamine (100 mg/kg, KETASET^®^, Zoetis US LLC, Parsippany, NJ, USA) and xylazine (10 mg/kg, Cronus Pharma, East Brunswick, NJ, USA) prepared in phosphate-buffered saline (PBS). Following confirmation of deep anesthesia, mice were transcardially perfused with PBS and subsequently fixed with 10% neutral buffered formalin (NBF, Sigma-Aldrich, St. Louis, MO, USA). Following perfusion, brains were dissected and fixed overnight in NBF at 4 °C. The tissues were then transferred to 30% sucrose in PBS and stored at 4 °C until fully cryoprotected, as indicated by sinking to the bottom of the vial. Brains were subsequently embedded in OCT compound, frozen, and stored on dry ice. Coronal sections (30 μm thick) were prepared using a TN50 cryostat (Tanner Scientific^®^, Sarasota, FL, USA). Unbiased stereology has been used for this study: systematic random sampling across DG and LV contour; for neurogenesis quantification, 6 serial coronal sections covering the full dentate gyrus were analyzed for each mouse. For each section, five non-overlapping confocal fields within the SGZ or SVZ of both left and right hemispheres were captured. Immunopositive cells were blindly quantified. Each group included 3 wild-type and 4 KO mice.

IF was performed as previously described [22]. Confocal images were acquired using a Leica TCS SP8 confocal microscope. Quantitative analysis of IF signal in confocal images was performed using the Leica Application Suite X (LASX, version 4.7.0) software and NIH-developed open software package ImageJ (V1.54r.). Positively stained cells in the studied hippocampal areas were manually selected and quantified with the ImageJ software. IF intensity quantification was analyzed with LAS X software.

### 4.3. BrdU Injection and Immunohistochemistry (IHC)

Three intraperitoneal injections (i.p.) of bromodeoxyuridine (BrdU, 50 mg/kg; Sigma Aldrich, St. Louis, MO, USA) were administered to 10-month-old mice at intervals of 24 h. The injected mice were perfused 2 weeks later as described above for brain sectioning. IHC of BrdU on the brain sections then were provided by the MHIR Histology Core following standard IHC protocol.

### 4.4. Antibodies

Immunofluorescence staining was performed using primary antibodies at a final concentration of 2 μg/mL. Primary antibodies included anti-RBFOX3 (NeuN; Thermo Fisher Scientific, Waltham, MA, USA, Cat. #PA5-78639), anti-DCX (Proteintech, Rosemont, IL, USA, Cat. #13925-1-AP), anti-IBA1 (Cell Signaling Technology, Boston, MA, USA, Cat. #17198), anti-GFAP (Cell Signaling Technology, Cat. #12389S), anti-OLIG2 (Cell Signaling Technology, Cat. #65915S), and anti-BrdU (MP Biomedical, SKU:0811200 and staining service provided by the MHIR Histology Core, Scarborough, ME, USA). The following secondary antibodies were used for immunofluorescence staining at a dilution of 1:250: AffiniPure™ Fab Fragment Goat Anti-Rabbit IgG (Fc fragment-specific) conjugated to Alexa Fluor^®^ 488 (Jackson ImmunoResearch Laboratories, West Grove, PA, USA, Cat. #111-547-008) and AffiniPure™ Fab Fragment Goat Anti-Rabbit IgG (Fc fragment-specific) conjugated to Alexa Fluor^®^ 647 (Jackson ImmunoResearch Laboratories, Cat. #111-607-008). For immunohistochemistry, Biotin-SP-conjugated Goat Anti-Mouse IgG (H + L) secondary antibody (Jackson ImmunoResearch Laboratories, Cat. #115-066-072) was used at a dilution of 1:500.

### 4.5. Statistics

Statistical analyses were performed using GraphPad Prism 11.0. Unless otherwise stated in the figure legends, data are presented as the mean ± SEM. For two-group comparisons, normally distributed data were analyzed using unpaired or paired Student’s *t*-test. For comparisons among multiple groups, two-way ANOVA followed by Tukey’s post hoc test was performed when the data satisfied the assumptions of parametric analysis.

## 5. Conclusions

Our findings demonstrate that DIO3-mediated T3 homeostasis is essential for maintaining ANG in the female brain. Importantly, partial rescue of the neurogenic deficit phenotype in *Dio2/Dio3* double KO mice provides further evidence supporting the idea that excessive T3 signaling may causally contribute to these defects. Together, these findings support a role for DIO3 in ANG and suggest that local T3 homeostasis affects this process via sexually dimorphic mechanisms.

## Figures and Tables

**Table 1:** Fold changes in thyroid hormone–responsive genes in the cortex of 15-month-old *Dio3*KO mice determined by RNA-seq. (*n* = 6 WT male, 6 KO male, 6 WT female, 6 KO female).

Gene Symbol	Sex	Log2Fold Changes Compare to WT	*p*-Value	Significance	Functional Annotation/Description
***Atoh8***	M	0.222	0.3199	-	Regulates neuronal lineage specification and differentiation [55]
	F	0.338	0.0410	*	
***Ccdc80*** **△**	M	−0.259	0.0401	*	Extracellular matrix protein regulating cell adhesion [56]
	F	0.338	0.0410	*	
***Emx2***	M	−0.209	0.17847	-	Controls neural progenitor proliferation and differentiation [57]
	F	−0.576	1.6 × 10^−5^	****	
***Esr1*** **△**	M	−0.083	0.7557	-	Regulates neurogenesis, neuronal survival, and estrogen signaling [58]
	F	0.917	1.34 × 10^−5^	****	
***Foxp1*** **△**	M	0.030	0.8470	-	Regulates neural progenitor proliferation and differentiation [59,60]
	F	−0.542	3.34 × 10^−6^	****	
***Hr***	M	0.477	0.0001	***	Modulates thyroid hormone receptor-mediated transcription [61]
	F	0.480	0.0001	***	
***Jun***	M	0.023	0.86018	-	AP-1 transcription factor regulating cell proliferation [62]
	F	0.553	1.8 × 10^−5^	****	
***Klf9***	M	0.277	0.0009	***	TH-responsive transcription factor regulating differentiation [63]
	F	0.197	0.0470	*	
***Nr2e1***	M	−0.214	0.0116	*	Controls neural stem cell proliferation and neurogenesis [64]
	F	−0.121	0.1690	-	
***Sparc***	M	0.463	1.1 × 10^−7^	****	Matricellular protein regulating extracellular matrix remodeling [65]
	F	0.298	0.0002	***	
***Thrb***	M	−0.119	0.2114	-	Regulates thyroid hormone-dependent gene expression [66,67]
	F	−0.191	0.0097	**	
***Tnc*** **△**	M	−0.480	0.0124	*	Modulates stem cell niche and neurogenesis [68]
	F	0.102	0.5977	-	

△ indicates opposite-sex responses. * *p* < 0.05, ** *p* < 0.01, *** *p* < 0.001, **** *p* < 0.0001 versus control group.

## Data Availability

The original contributions presented in this study are included in the article/Supplementary Material. Further inquiries can be directed to the corresponding author.

## Supplementary Materials

The following supporting information can be downloaded at https://www.mdpi.com/article/10.3390/ijms27177607/s1.

## Abbreviations

The following abbreviations are used in this manuscript:

ANG	Adult neurogenesis
Atoh8	Atonal BHLH Transcription Factor 8
CALB1	Calbindin 1
Ccdc80	Coiled-Coil Domain Containing 80
DAPI	4′,6-diamidino-2-phenylindole
DCX	Doublecortin
*Dio2*	Deiodinase 2
*Dio3*KO	Deiodinase 3-deficient
Emx2	Empty Spiracles Homeobox 2
Esr1	Estrogen Receptor 1
GFAP	Glial fibrillary acidic protein
Foxp1	Forkhead Box P1
Hr	Hairless
IBA1	Ionized calcium binding adaptor molecule 1
Jun	Jun Proto-Oncogene
Klf9	KLF Transcription Factor 9
LV	Lateral ventricle
Nr2e1	Nuclear Receptor Subfamily 2 Group E Member 1
OLIG2	Oligodendrocyte Transcription Factor 2
*Pink1*	PTEN Induced Kinase 1
PTEN	Phosphatase And Tensin Homolog
RBFOX3	RNA-binding fox-1 homolog 3
Sparc	Secreted Protein Acidic And Cysteine Rich
SVZ	Subventricular zone
TH	Thyroid hormone
THR	Thyroid hormone receptor
Thra	Thyroid Hormone Receptor Alpha
Thrb	Thyroid Hormone Receptor Beta
Tnc	Tenascin C
