# Supplementary material for: Deiodinase 3 Deficiency Impairs Hippocampal and Lateral Ventricle Neurogenesis in Middle-Aged Female Mice
Source: Int J Mol Sci. 2026 Aug 25;27(17):7607. doi: 10.3390/ijms27177607 (PMC13566596; doi:10.3390/ijms27177607)
Supplement: Supplementary file 1 [file ijms-27-07607-s001.zip › ijms-4470868-supplementary.pdf]

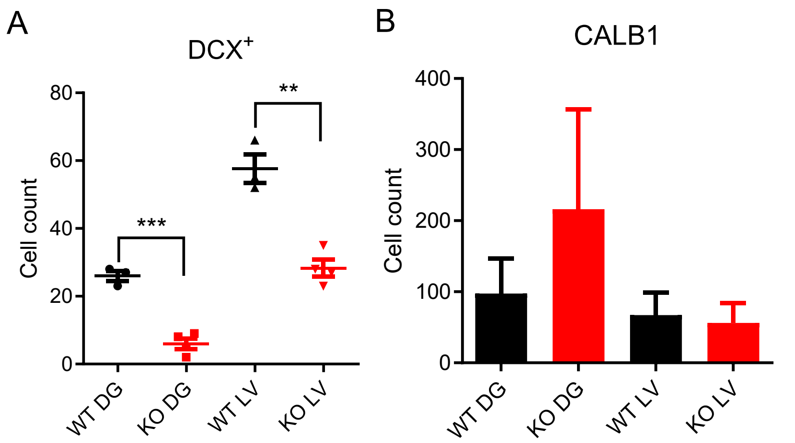

Figure S1.Statistics of DCX+ and CALB1+ cells in the brains of 10 MO female *Dio3*KO mice. A, quantification of DCX-positive cells; B, CALB1-positive cells in the regions of interest. DG, dentate gyrus; LV, lateral ventricle. **, p<0.01,***, p<0.001, by Student's t-test. N=3.4.

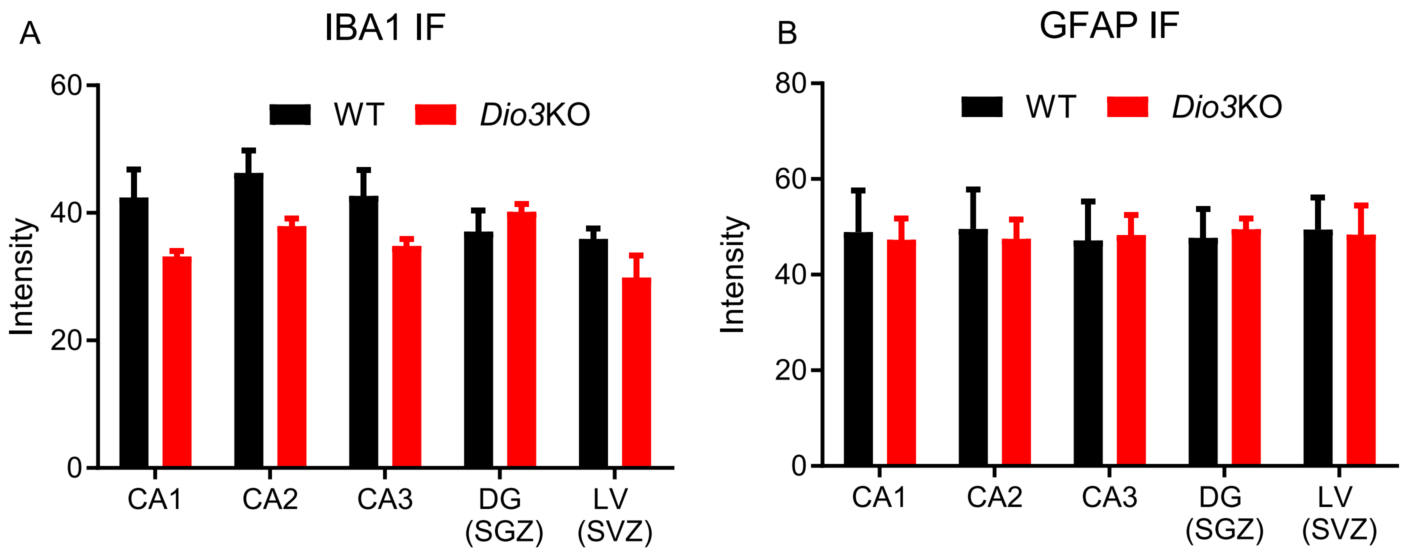

Figure S2.Statistics of IBA1, GFAP immunofluorescence intensity in the brain of female *Dio3*KO mice. A, quantitative analysis of IBA1; B, quantitative analysis of GFAP. N=3.4. CA1-3: three subfields of the Cornu Ammonis (the hippocampus proper); DG, dentate gyrus; LV, lateral ventricle.

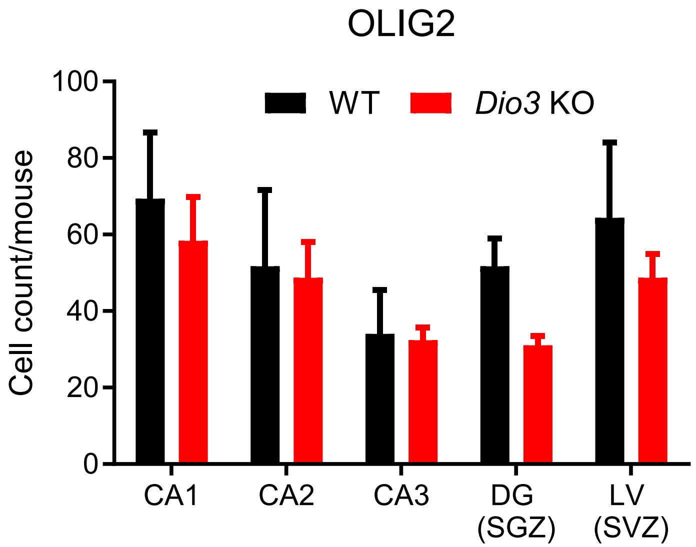

Figure S3-1 Statistics of OLIG2+ cells in the brain of female *Dio3* KO mice. N= 3,4. CA1-3: three subfields of the Cornu Ammonis (the hippocampus proper); DG, dentate gyrus; LV, lateral ventricle.

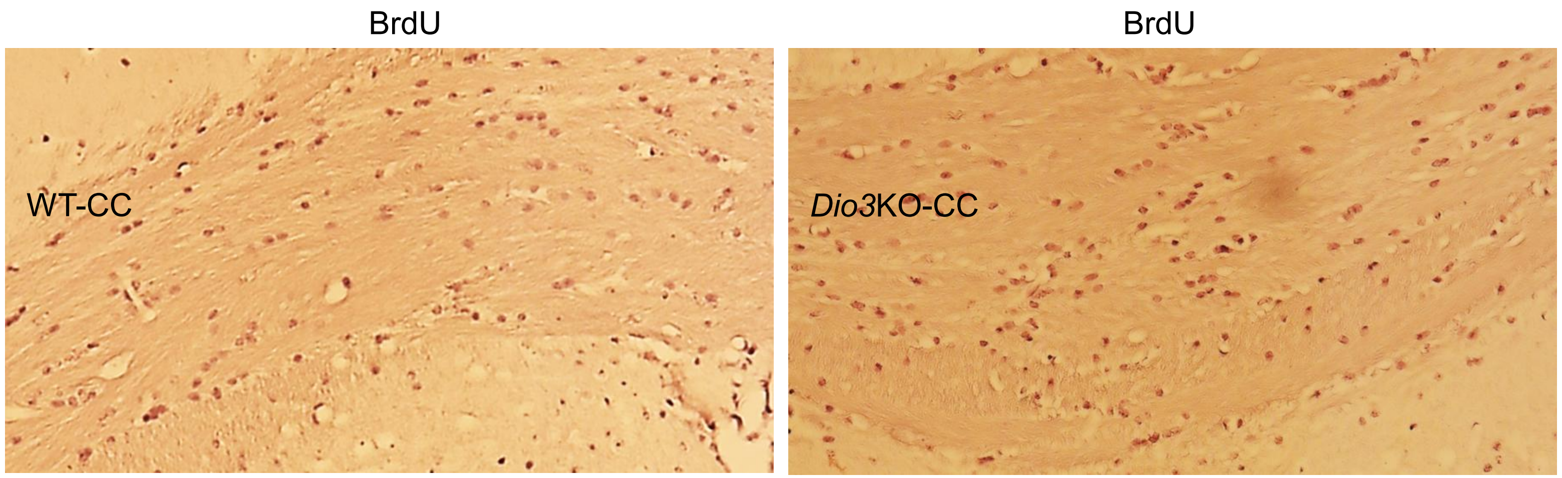

Figure S3-2. Immunohistochemistry BrdU Staining in the Corpus Callosum (CC) of 10 month-old male *Dio3*KO Mouse Brains (AP: Bregma −0.9 mm; ML: midline +1 mm).

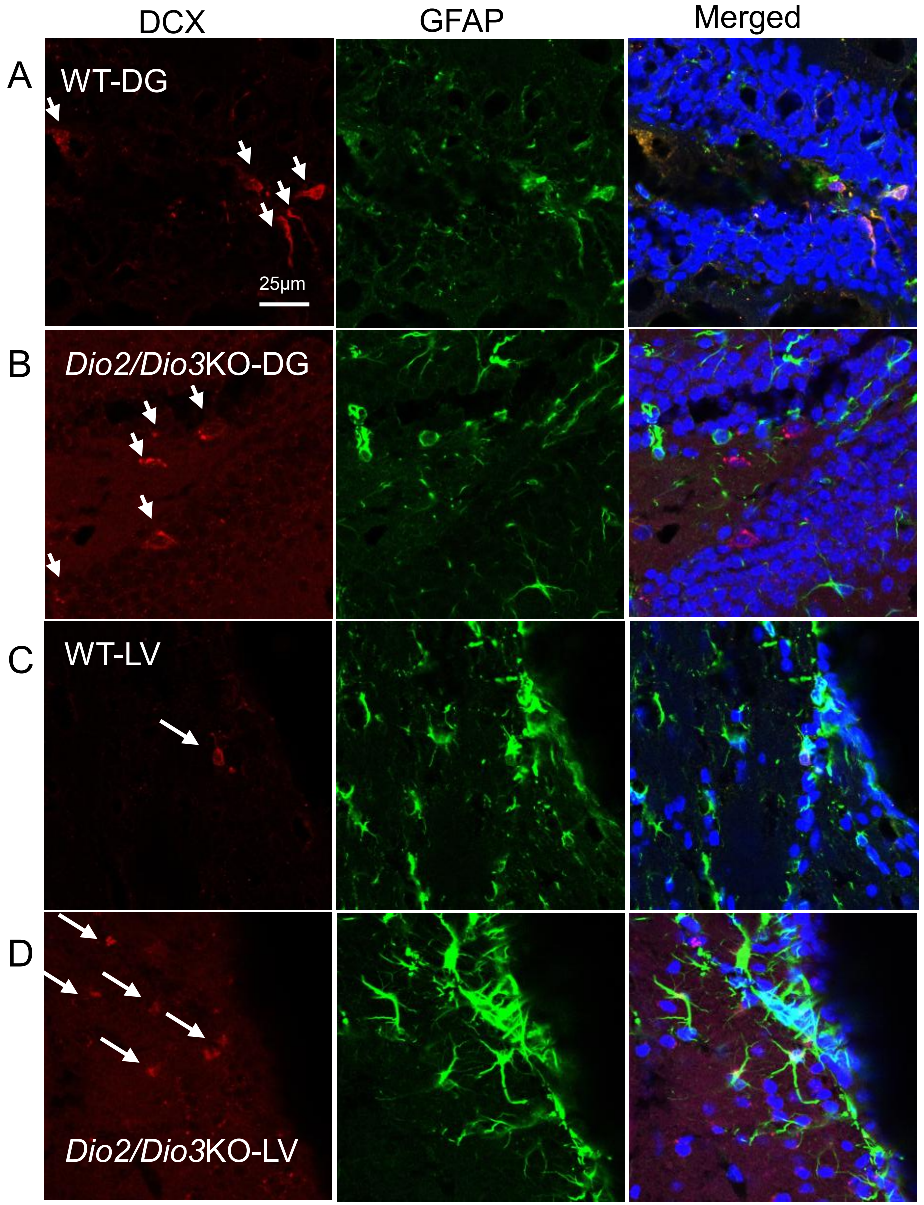

Figure S4. DCX and GFAP double staining in male *Dio2/Dio3* double KO Mouse Brains. (A, B) Representative images of DCX/GFAP double immunofluorescence staining in the SGZ of the dentate gyrus. (C, D) Representative images of DCX/GFAP double immunofluorescence staining in the SVZ of the lateral ventricle. DCX staining is shown in red, GFAP staining in green, and DAPI nuclear staining in blue. DG, dentate gyrus; LV, lateral ventricle.
